# Large Scale Immune Profiling of Infected Humans and Goats Reveals Differential Recognition of *Brucella melitensis* Antigens

**DOI:** 10.1371/journal.pntd.0000673

**Published:** 2010-05-04

**Authors:** Li Liang, Diana Leng, Chad Burk, Rie Nakajima-Sasaki, Matthew A. Kayala, Vidya L. Atluri, Jozelyn Pablo, Berkay Unal, Thomas A. Ficht, Eduardo Gotuzzo, Mayuko Saito, W. John W. Morrow, Xiaowu Liang, Pierre Baldi, Robert H. Gilman, Joseph M. Vinetz, Renée M. Tsolis, Philip L. Felgner

**Affiliations:** 1 Division of Infectious Diseases, Department of Medicine, University of California Irvine, Irvine, California, United States of America; 2 Alexander von Humboldt Institute of Tropical Medicine, Universidad Peruana Cayetano Heredia, Lima, Peru; 3 Department of Computer Science, University of California Irvine, Irvine, California, United States of America; 4 Institute for Genomics and Bioinformatics, University of California Irvine, Irvine, California, United States of America; 5 Department of Medical Microbiology and Immunology, University of California Davis, Davis, California, United States of America; 6 Department of Veterinary Pathobiology, Texas A&M University and Texas Agricultural Experiment Station, College Station, Texas, United States of America; 7 Asociacion Benefica PRISMA, Lima, Peru; 8 Antigen Discovery, Inc., Irvine, California, United States of America; 9 Department of Biological Chemistry, University of California Irvine, Irvine, California, United States of America; 10 Department of International Health, Bloomberg School of Public Health, Johns Hopkins University, Baltimore, Maryland, United States of America; 11 Division of Infectious Diseases, University of California San Diego School of Medicine, La Jolla, California, United States of America; Swiss Tropical Institute, Switzerland

## Abstract

Brucellosis is a widespread zoonotic disease that is also a potential agent of bioterrorism. Current serological assays to diagnose human brucellosis in clinical settings are based on detection of agglutinating anti-LPS antibodies. To better understand the universe of antibody responses that develop after *B. melitensis* infection, a protein microarray was fabricated containing 1,406 predicted *B. melitensis* proteins. The array was probed with sera from experimentally infected goats and naturally infected humans from an endemic region in Peru. The assay identified 18 antigens differentially recognized by infected and non-infected goats, and 13 serodiagnostic antigens that differentiate human patients proven to have acute brucellosis from syndromically similar patients. There were 31 cross-reactive antigens in healthy goats and 20 cross-reactive antigens in healthy humans. Only two of the serodiagnostic antigens and eight of the cross-reactive antigens overlap between humans and goats. Based on these results, a nitrocellulose line blot containing the human serodiagnostic antigens was fabricated and applied in a simple assay that validated the accuracy of the protein microarray results in the diagnosis of humans. These data demonstrate that an experimentally infected natural reservoir host produces a fundamentally different immune response than a naturally infected accidental human host.

## Author Summary

Brucellosis is a bacterial disease transmitted from infected animals to humans. This disease often presents as a prolonged but non-specific illness primarily characterized as fever without specific organ localization. Because infections can result after ingestion (typically from unpasteurized animal milk or milk products from goats, cattle or sheep) or inhalation (important because of bioterrorism potential) of small numbers of organisms, the bacteria that cause brucellosis are potential biological warfare agents. Here, a protein microarray containing 1406 *Brucella melitensis* proteins was used to study the antibody response of experimentally infected goats and naturally infected humans in *B. melitensis* infection. Goats recognized 18 proteins and humans recognized 13 proteins as serodiagnostic antigens; antibody detection of only two of these antigens was shared by goats and humans, suggesting either fundamentally different immune responses or different responses in relation to mode or setting of infection. The human serodiagnostic antigens were evaluated in a simple nitrocellulose line blot assay, which validated the protein microarray results. The approach described here will lead to the development of new diagnostics for brucellosis and other infectious diseases, and aid in understanding the human and animal host immune response to pathogenic organisms.

## Introduction

Brucellosis is a zoonotic infectious disease endemic in regions around the world where agricultural, animal husbandry and vaccination practices have not controlled infection among livestock reservoirs [1]–[3]. The reservoirs of *Brucella melitensis*, the most virulent species affecting humans, include goats and sheep [4], especially in Peru and the Middle East [3]. Identification of goat, sheep and other animal sources of infection have long used agglutination tests, although newer tests are being developed and applied in the veterinary setting [5]–[7]. Commonly used screening tests do not necessarily differentiate between vaccination and infection in goats ([8]; summarized in [6]). By themselves, the Rose Bengal and other agglutination tests cannot be used exclusively to diagnose human brucellosis because while sensitive and specific for first episodes of brucellosis, these tests can be problematic in differentiating acute, chronic and relapsing forms of brucellosis in humans living in endemic regions [9]–[12], and typically require titration and differentiation of IgM from IgG antibodies either in solid phase formats or by use of the mercaptoethanol test [1], [3], [13]–[16].

The current knowledge of protein antigens recognized by humans and reservoir animals is limited to a relatively small number of immunogenic *Brucella abortus* proteins recognized by cattle, mice and sheep and limited studies on human and goat recognition of *Brucella melitensis* antigens [9]–[11], [17]–[33]. No individual antigen has proven to be of sufficient diagnostic utility to replace the LPS-based tests. Indeed, antibodies to smooth LPS have been observed to arise sooner in the course of brucellosis compared to known antigens or groups of uncharacterized cytoplasmic protein antigens [15], [34]–[43], especially if treatment is initiated early after clinical presentation [43]. We tested the hypothesis that the immune response to *B. melitensis* infection of natural reservoir host (goat) and accidental host (humans) is similar despite potentially different routes of infection. For this we constructed a protein microarray consisting of 1406 *B. melitensis* proteins and probed with a collection of sera from naturally infected and control human sera from Lima Peru, and goats experimentally infected with virulent *B. melitensis* 16M.

## Materials and Methods

### Ethics statement

Human sera were obtained from patients enrolled in a prospective clinical study of brucellosis in Lima, Peru. The human subjects part of the study was approved by the Humans Research Protections Committee of the University of California San Diego, the Comite de Ética of Universidad Peruana Cayetano Heredia, Lima, Peru and the Comite de Ética of Asociación Benéfica PRISMA, Lima, Peru, all of whom have maintained federal wide assurances with the United States Department of Health and Human Services. All patients provided written informed consent prior to enrollment in the study, and signed consent forms have been stored in locked files in study offices at UPCH and AB PRISMA, Lima, Peru.

Goat sera were obtained from previously stored samples from experimentally infected goats under Institutional Animal Care and Use protocols approved by Texas A&M University, College Station, Texas, USA. Animals were housed in an outdoor, restricted access, large-animal isolation facility operated under guidelines approved by the United States Department of Agriculture/Animal and Plant Health Inspection Service (USDA/APHIS). At the termination of the experiments, adult animals were euthanized by captive bolt. All animals were disposed of by University approved protocols.

### Gene amplification and cloning

Genes were amplified and cloned using high-throughput PCR and recombination method as described previously [44]. ORFs from *Brucella melitensis* 16M genomic DNA were identified using GenBank NC_003317 and NC_003318, amplified using gene specific primers containing 33bp nucleotide extension complementary to ends of linearized pXT7 vector. Homologous recombination takes place between the PCR product and pXT7 vector in competent DH5a cells. The recombinant plasmids were isolated from this culture using QIAprep 96 Turbo kit (Qiagen). Around one quarter of the cloned genes were sequenced and verified that the correct sequence was inserted. The resulting fusion proteins also harbor a hemagglutinin epitope at 3′ end and polyhistidine at the 5′ end.

### Microarray printing and staining

Plasmids were expressed at 30°C in 5 hour- *in vitro* transcription/translation *E. coli* system (RTS 100 kits from Roche), according to the manufacturer's instructions. For microarrays, 15 µl of reaction was mixed with 5 µl 0.2% Tween 20 to give a final concentration of 0.05% Tween 20, and 15-µl volumes were transferred to 384-well plates and printed onto nitrocellulose coated glass FAST slides (Whatman) using Omni Grid 100 microarray printer (Genomic Solutions). Protein expression and printing was monitored by immunoprobing with anti-polyhistidine (clone His-1, Sigma) and anti-hemagglutinin (clone 3F10, Roche). For all array staining, sera samples were diluted to 1∶200 in Protein Array Blocking Buffer (Schleicher & Schuell). Slides were first blocked for 30 min in protein array-blocking buffer before incubation with primary antibody at 4°C overnight with agitation. The slides are then washed extensively and incubated in biotin-conjugated secondary antibody (Jackson Immuno Research) diluted 1/200 in blocking buffer. After washing, bound antibodies are detected by incubation with streptavidin-conjugated SureLight® P-3 (Columbia Biosciences). The slides are washed and air dried after brief centrifugation. Slides were scanned and analyzed using a Perkin Elmer ScanArray Express HT microarray scanner. Intensities are quantified using QuantArray software. All signal intensities are corrected for spot-specific background.

### *Brucella melitensis* serum samples

Human sera tested in this study were obtained from the following patient groups: patients confirmed (by positive blood culture) to have acute brucellosis in Lima, Peru; from culture-negative, Rose Bengal-positive patients presenting with brucellosis-compatible syndromes; Rose Bengal-negative patients referred by their physicians for possible brucellosis; and ambulatory, apparently healthy control patients from the north Lima neighborhood of Puente Piedra where brucellosis is known to occur with risk factors similar to those in the rest of Lima. No patients in this study were known to be directly exposed to goats; risk factors for all were reported to be ingestion of unpasteurized goat's milk products, the typical risk factor in Lima for acquisition of brucellosis. All patients included in this study had their first known episode of brucellosis, with clinical presentation within 1–3 weeks of onset of symptoms. The patient samples were as follows: 42 serum samples *from B. melitenis* culture-positive patients all of whom were positive by the Rose Bengal screening test and had tube agglutination tests > = 1/160; and 18 samples from culture negative, Rose Bengal serology-positive patients. These latter 18 samples were from culture negative individuals diagnosed with brucellosis and treated according to standard antibiotic therapy within 2 days of serum sampling. Additional control patient samples included 13 sera from Rose Bengal-negative patients, 44 samples from ambulatory healthy controls from north Lima where brucellosis occasionally affects patients, and sera from humans in the U.S. where brucellosis is not found.

Goat sera tested in this study were positive (*B. melitensis* 16M-infected) and negative (uninfected) controls from a previously conducted vaccine safety study [23] in which pregnant, card-test negative angora goats were inoculated with *B. melitensis*. Goats were experimentally infected with 1×10^7^ CFU of *Brucella melitensis* strain 16M by bilateral conjunctival instillation at 110 days' gestation, and sera were collected 8 weeks after infection. As an additional negative control, 15 serum samples from a specific pathogen-free goat flock were obtained (Capralogics, Inc, Hardwick, MA).

### Immunostrip printing and probing

Thirteen plasmids of interest were expressed in five hour *in vitro* transcription-translation reactions (RTS 100 E. coli HY Kit from Roche) according to the manufacturer's instructions. VIG was obtained from ADi as a gift, and the concentration of VIG was diluted to 0.05 mg/ml. Proteins were printed on Optitran BA-S 85 0.45 µm Nitrocellulose membrane (Whatman) using BioJet dispenser (BioDot) at 1 µl/cm, and cut into 3 mm strips. Individual strips were then blocked in 10% non fat dry milk dissolved in 10 mM Tris (pH 8.0) and 150 mM NaCl containing 0.05% (v/v) Tween 20 buffer for 30 minutes. Prior to immunostrip probing, forty two culture positive and forty four Peruvian naive sera were diluted to 1/250 in 10% non fat dry milk solution containing 20% *E. coli* lysate (McLab) and incubated for 30 minutes with constant mixing at room temperature. Each strip was then incubated with pretreated sera overnight at 4°C with gentle mixing. Strips were then washed five times in Tris buffer containing 0.05% (v/v) tween 20, and then incubated for 1 hour at room temperature in alkaline phosphatase conjugated donkey anti-human immunoglobulin (anti-IgG, Fcγ fragment-specific, Jackson ImmunoResearch), diluted to 1/5000 in tris buffer containing 0.05% (v/v) tween 20. Strips were then washed extensively and reactive bands were visualized by incubating with 1-step Nitro-Blue Tetrazolium Chloride/5-Bromo-4-Chloro-3′-Indolyphosphate p-Toluidine Salt (NBT/BCIP) developing buffer (Thermo Fisher Scientific) for 1 minute at room temperature. Strips were scanned with Hewlett-Packard scanner, and were quantified using Image J software.

### Data analysis

All analysis was performed using the R statistical environment (http://www.r-project.org). It has been noted in the literature that data derived from microarray platforms is heteroskedatic [45]–[48]. This mean-variance dependence has been observed in the arrays presented in this manuscript [49], [50]. In order to stabilize the variance, the vsn method [51] implemented as part of the Bioconductor suite (www.bioconductor.org) is applied to the quantified array intensities. In addition to removing heteroskedacity, this procedure corrects for non-specific noise effects by finding maximum likelihood shifting and scaling parameters for each array such that control probe variance is minimized. This calibration has been shown to be effective on a number of platforms [52]–[54]. Normalized data is retransformed with the ‘sinh’ function to allow visualization and discussion at an approximate raw scale.

Diagnostic biomarkers between groups were determined using a Bayes regularized t-test adapted from Cyber-T for protein arrays [47], [48], which has been shown to be more effective than other differential expression techniques [55]. To account for multiple testing conditions, the Benjamini and Hochberg (BH) method was used to control the false discovery rate [56]. Multiplex classifiers were constructed using linear and non-linear Support Vector Machines (SVMs) using the “e1071” R package. SVM is a supervised learning method that has been successfully applied to microarray data characterized by small samples sizes and a large number of attributes [50], [57]. The SVM approach, as any other supervised classification approach, uses a training dataset to build a classification model and a testing set to validate the model. To generate unbiased training and testing sets, leave one out cross-validation (LOOCV) was used. With this methodology, each data point is tested with a classifier trained using all of the remaining data points. Plots of receiver operating characteristic (ROC) curves were made with the ‘ROCR” R package.

## Results

### Gene amplification, cloning and protein expression

**Figure 1:**
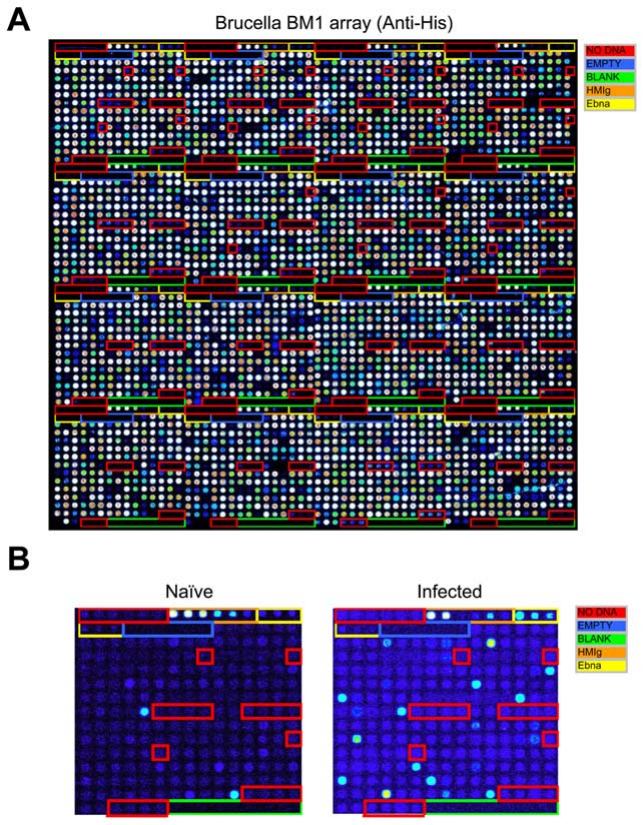
Construction of a *B. melitensis* Protein Microarray. Arrays were printed containing 1406 *B. melitensis* proteins, positive and negative control spots. Proteins were printed in duplicates. Each array contains positive control spots printed from 6 serial dilutions of human and mouse IgG, 6 serial dilutions of EBNA1 protein, and “No DNA” negative control spots. (A) The array was probed with anti-His antibody as described in Materials and Methods, to confirm the expression and printing of over 95% proteins. (B) Comparison of arrays probed with Peruvian naïve serum and Culture positive serum. The arrays were read in a laser confocal scanner, analyzed, and the data normalized as described in Materials and Methods. The signal intensity of each antigen is represented by rainbow palette of blue, green, red and white by increasing signal intensity.

A set of 1406 ORFs from *Brucella melitensis* 16M was selected for this study. We picked 1009 antigens based on their Psort information and B cell epitope prediction score, and 397 ORFs were randomly selected. The ORFs were amplified from *Brucella melitensis* 16M (*Bm*) genomic DNA and cloned using the high throughput recombination method previously described [44]. About one-fourth of the cloned genes were sequenced and >99% of sequenced clones had the correct sequence in frame with correct orientation. *Bm* ORFs cloned in pXT7 vector were expressed under T7 promoter in the *E. coli in vitro* transcription/translation system, and printed in duplicates on microarrays as described in Methods and 97.4% of the proteins were positive for the His tag (**Fig. 1a**), and 95.4% were positive for HA tags.

### Immune screening with goat serum samples

**Figure 2:**
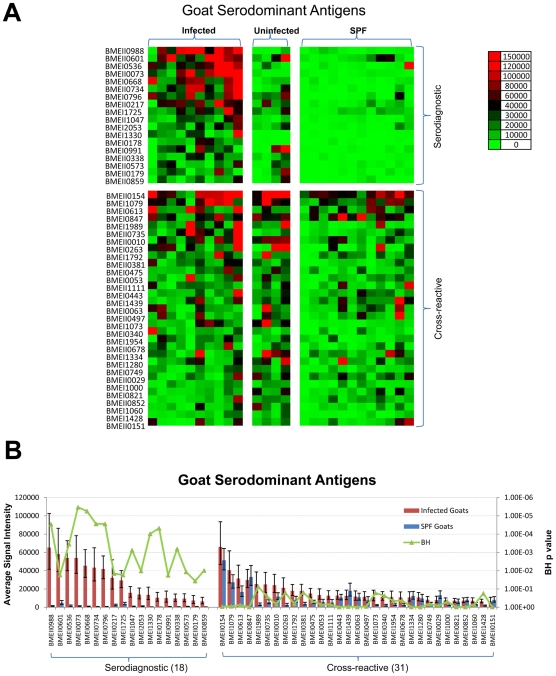
Probing a collection of *B. melitensis* infected, uninfected, and SPF control goat sera and discovery of goat serodiagnostic antigens. Arrays containing 1406 *B. melitensis* proteins were probed with goat sera organized into 3 groups as described in the text. (A). Heatmap showing normalized intensity with red strongest, bright green weakest and black in between. The antigens are in rows and are grouped to serodiagnostic and cross-reactive antigens. The goat samples are in columns and sorted left to right by increasing average intensity to serodiagnostic antigens. (B) The mean sera reactivity of the 1406 antigens was compared between the Infected and SPF Naive groups. Antigens with Benjamini Hochberg corrected p-value less than 0.05 are organized to the left and cross-reactive antigens to the right. The 18 most reactive serodiagnostic and 31 of the most reactive cross-reactive antigens are shown.

*Bm* protein arrays were probed with sera from experimentally infected goats, naïve goats from the same pasture, and specific pathogen free (SPF) goats from a different location. Reactivity of sera from the individual goats is shown as a heat map with samples grouped according to their description (**Fig. 2a**). Data were analyzed using methods described elsewhere [58]. Serodominant antigens are defined as antigens with mean signal intensity greater than the mean plus two standard deviations above the negative controls. Serodiagnostic antigens are significantly differentially reactive serodominant antigens with adjusted Cyber-T p-values between infected and SPF goats <0.05. All of the sera, whether from infected, uninfected or naïve goats, reacted similarly to the cross-reactive antigens (p-value >0.05). A set of 49 antigens were identified to be serodominant among 1406 antigens tested. Of these, 18 antigens were serodiagnostic, and reacted differentially between infected goats and SPF goats (p-value <0.05). The remaining 31 serodominant antigens reacted similarly among all goats (**Fig. 2a, 2b**).

### Human antigenic profile

**Figure 3:**
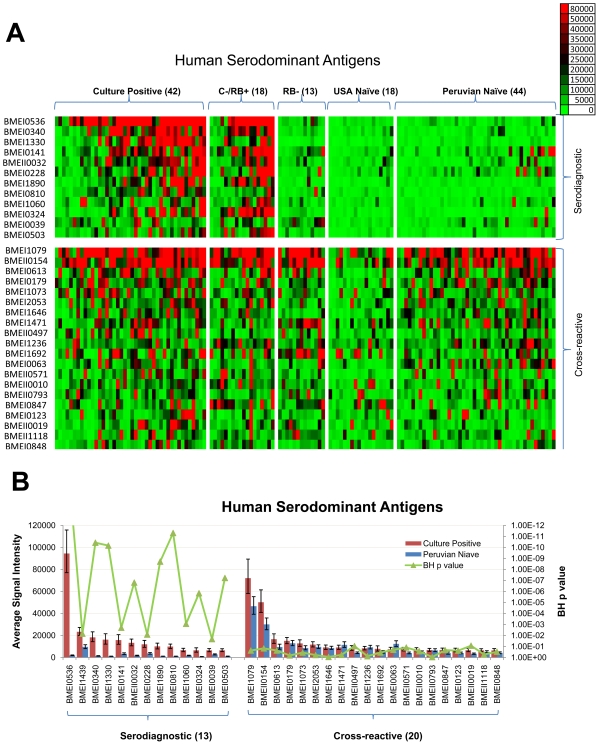
Probing a collection of *B. melitensis* human sera and discovery of human serodiagnostic antigens. Arrays were probed with human sera organized into 5 groups: Culture Positive, Culture Negative/Rose Bengal Positive, Rose Bengal Negative, USA Naïve, and Peruvian Naïve, as described in the text. (A). Heatmap showing normalized intensity with red strongest, bright green weakest and black in between. The antigens are in rows and are grouped to serodiagnostic and cross-reactive antigens. The human samples are in columns and sorted left to right by increasing average intensity to serodiagnostic antigens. (B) The mean sera reactivity of the 1406 antigens was compared between the Culture Positive and Peruvian Naive groups. Antigens with Benjamini Hochberg corrected p-value less than 0.05 are organized to the left and cross-reactive antigens to the right. The 13 most reactive serodiagnostic and 31 of the most reactive cross-reactive antigens are shown. C−/RB+, Culture Positive and Rose Bengal negative; RB−, Rose Bengal negative. Numbers in () are case numbers from each group.

*Bm* protein arrays were also probed with sera from acute brucellosis patients in Lima, Peru obtained within 1–3 weeks of the onset of symptoms. All patients in this study, as is true of virtually all patients from Lima [3], [59]–[62], were infected with *B.. melitensis* biovar 1. Sera from *Bm* culture-positive humans (**Fig. 1b**) showed pronounced reactivity against several antigens compared to unexposed individuals. A set of 33 antigens was identified to be serodominant among 1406 antigens tested (**Fig. 3a, 3b**). Of these, 13 antigens were serodiagnostic, and reacted differentially between naïve and culture positive patients from Peru (p-value<.05). The same antigens also reacted robustly with individuals diagnosed Rose Bengal positive but negative by blood culture for *B. melitensis*. For some of these subjects, treatment with antibiotics may have resulted in a negative blood culture for *B. melitensis*. The elevated antibody response from a few individuals in the Peruvian naïve group might be indicative of past exposure to similar proteins in environmental bacteria, or to a past subclinical *Brucella* infection. We also identified 20 cross-reactive antigens that reacted similarly among all human samples, whether from naïve individuals or individuals diagnosed to be infected and use of these antigens in serodiagnostic tests can therefore be selectively avoided.

### Identification of serodiagnostic antigens

**Figure 4:**
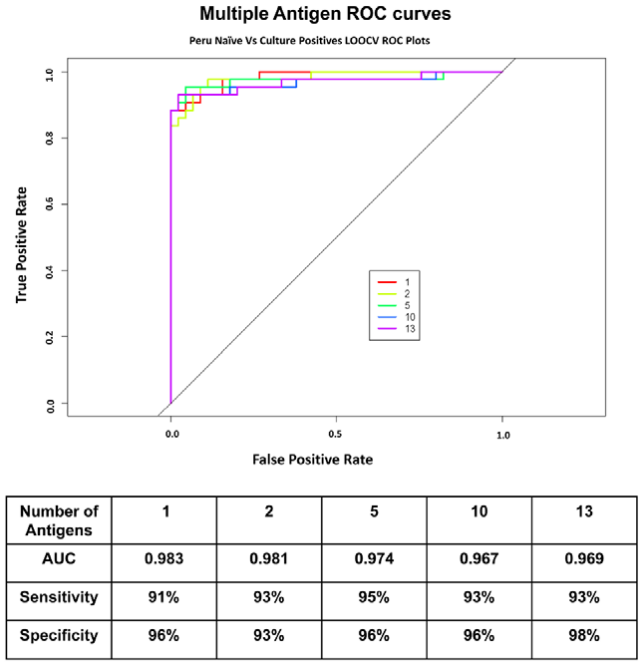
Multiple Antigen LOOCV ROC curves. The LOOCV ROC graphs show classifiers with increasing number of human serodiagnostic antigens. Overall, the sensitivity and specificity for array test is over 95%.

To establish a collection of antigens able to accurately distinguish brucellosis cases from controls, leave one out cross-validation (LOOCV) receiver operating characteristic (ROC) curves were generated for individual serodiagnostic antigens to assess the ability to separate the control and disease cases (**Fig. 4**). The serodiagnostic antigens were ordered by decreasing single antigen area under the curve (AUC). The top ten ORFs all have an AUC greater than 0.734 (Table 1), with BP26 (BMEI0536; AUC 0.983; Benjamini and Hochberg adjusted Cyber-T p-value<10e-16) giving the best single antigen discrimination with sensitivity and specificity 91% and 96% (**Fig. 4**), respectively. We used kernel methods and support vector machines [47], [63] to build linear and nonlinear classifiers. As input to the classifier, we used the highest-ranking 1, 2, 5, 10, 13 ORFs on the basis of single antigen AUC. The results show that increasing the antigen number from 2 to 5 produced an improvement in sensitivity and specificity (**Fig. 4**). This classifier yielded a high sensitivity and specificity rate of 95% and 96%, respectively.

**Table 1:** Common and specific *Brucella melitensis* antigens for humans and goats.

	*B. melitensis 16M*	*B. abortus 2308*	*B. suis 1330*	AUC	Product Name	Reference for other *Brucella* species
**Serodiagnostic for both Humans and Goats**	BMEI0536	BAB1_1494	BR1475	0.983	26 kDa periplasmic immunogenic protein bp26	{Cloeckaert, 1996; Connolly, 2006; Lindler, 1996; Yang, 2005}
	BMEI1330	BAB1_0635	BR0611	0.870	Protease Do	{Roop, 1994}
**Serodiagnostic for Humans only**	BMEI1439	BAB1_0522	BR0497	0.715	Chromosome Segregation	
					Protein SMC	
	BMEI0340	BAB1_1707	BR1695	0.889	Omp16 lipoprotein	{Tibor, 1994}
	BMEI0141	BAB1_1922	BR1922	0.734	2-oxoglutarate dehydrogenase, E2 component, dihydrolipoamide succinyltransferase	
	BMEII0032	BAB2_0061	BRA0062	0.849	VirB8	
	BMEI0228	BAB1_1830	BR1822	0.719	Hypothetical protein	
	BMEI1890	BAB1_0051	BR0054	0.866	Transporter	
	BMEI0810	BAB1_1199	BR1177	0.918	COG1434 Uncharacterized conserved protein	
	BMEI1060	BAB1_0927	BR0909	0.763	DsbA Protein-disulfide isomerase	
	BMEI0324	BAB1_1726	BR1714	0.822	COG1360 Flagellar Motor Protein	
	BMEI0039	BAB1_2033	BR2032	0.700	Acetyl CoA carboxylase, carboxyltransferase, alpha subunit	
	BMEI0503	BAB1_1528	BR1510	0.865	COG1607 Acyl-CoA Hydrolase	
**Serodiagnostic for Goats only**	BMEII0988	BAB2_0943	BRA0260	0.942	Copper-containing nitrite reductase NirK	
	BMEII0601	BAB2_0558	BRA0682	0.917	ABC amino acid transporter, periplasmic binding protein	
	BMEII0073	BAB2_0019	BRA0020	1.000	Hypothetical protein	
	BMEI0668	BAB2_0441	BRA0797	1.000	Calcium binding protein Asp24	{Lin, 1995}
	BMEII0734	BAB2_0699	BRA0538	0.983	Oligopeptide binding protein precursor	
	BMEI0796	BAB1_0294	BR0263	0.950	TRAP transporter solute receptor Bcsp31	{Mayfield, 1988}
	BMEII0217	BAB2_1043	BRA1084	0.908	ABC dipeptide transport protein, periplasmic component	
	BMEI1725	BAB1_0226	BR0225	0.967	COG1732 glycine betaine-binding protein	
	BMEII1047	BAB2_0190	BRA0196	0.967	10kDa chaperonin groES	{Connolly, 2006}
	BMEI2053	BAB1_2075	BR2074	0.892	Calcium binding protein	
	BMEI0178	BAB1_1885	BR1885	0.967	Hypothetical protein	
	BMEI0991	BAB1_1009	BR0990	0.842	Rare Lipoprotein A	
	BMEII0338	BAB2_0275	BRA0960	0.958	ABC transporter periplasmic	
					BP, lipoprotein	
	BMEII0573	BAB2_0527	BRA0712	0.917	Transcriptional regulator, RpiR family	
	BMEII0179	BAB2_1078	BRA1120	0.858	Zn binding protein	
	BMEII0859	BAB2_0812	BRA0409	0.883	ABC dipeptide transport system, periplasmic component	

### Validation of serodiagnostic accuracy with immunostrips

**Figure 5:**
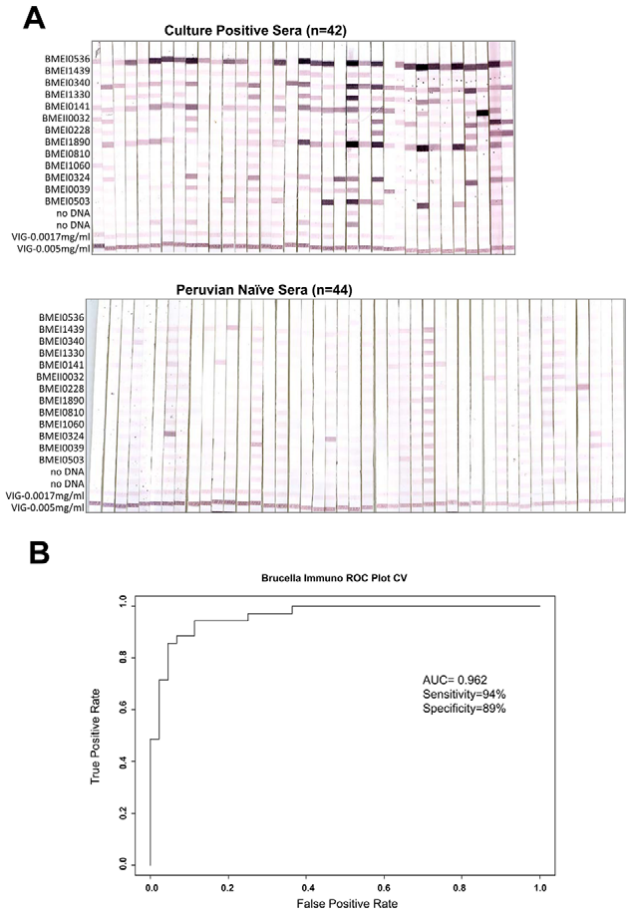
Immunostrips probing. (a)Thirteen serodiagnostic antigens were printed onto nitrocellulose paper in adjacent stripes using a BioDot jet dispenser as described in Materials and Methods. Strips were probed with Culture Positive or Peruvian naive sera diluted 1/200 followed by alkaline phosphatase conjugated secondary antibody and enzyme substrate. Weak reactivity in the naïve healthy controls can be distinguished from the strong reactivity in infected group. (b). The LOOCV ROC curve was generated and sensitivity and specificity of immunostrips probing test is 94% and 89%, respectively.

To test the feasibility of using the serodiagnostic antigens in an alternative analytical assay, thirteen serodiagnostic proteins were printed onto Nitrocellulose membranes using a BioDot jet dispenser. The paper was then cut into 3 mm strips (**Fig. 5a**). The individual strips were probed with 42 different culture positive sera and 44 Peruvian naive sera. Brucellosis patients reacted strongly against the serodiagnostic antigens with variable signal intensity among the patients. Naïve samples showed much lower reactivity against these serodiagnostic antigens. To assess the ability of antigens to separate disease and healthy controls, the LOOCV ROC curve was also generated (**Fig. 5b**). The ROC curve shows that this probing test yielded a high AUC of 0.962 with sensitivity rate of 94% and specificity rate of 89%. Thus, thirteen differentially reactive serodiagnostic antigens identified by microarray analysis in immunostrip format validated the list of serodiagnostic antigens to correctly classify *B. melitensis* positive sera.

The sensitivity and specificity of the top 5 serodiagnostic antigens discovered using the protein microarrays had sensitivity and specificity of 95% and 96%, better than that of the 13 antigens on the immunostrips (94% and 89%).

### Comparing antigenic proteins among humans and goats

**Figure 6:**
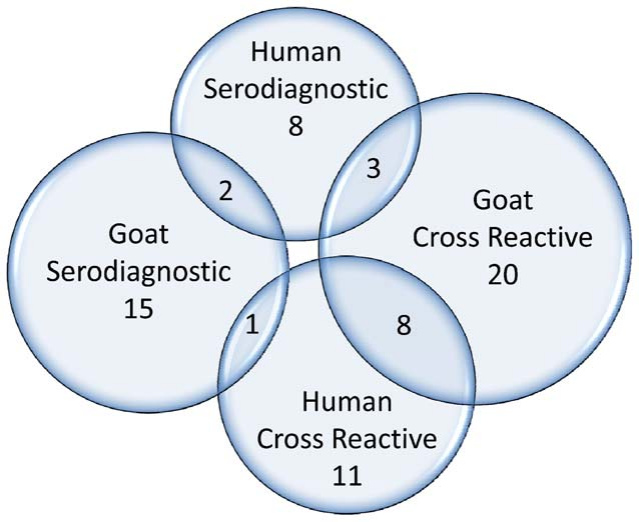
Serodiagnostic and cross-reactive antigens for humans and goats.

Both humans and animals can be infected by *Bm*. In the present study, goats were infected by *B. melitensis* strain 16M which would be expected to be virtually identical to the strains infected by patients in Lima given the limited diversity of the strains found there [3], [59]–[62]. To better understand the differences in the immune response to *Bm* infection between humans and goats, we compared serodominant antigens for both humans and goats. In the current study, two antigens are found to be serodiagnostic for both humans and goats (**Fig. 6**, **Table 1**). The top antigen on the list, BMEI0536 (Bp26 protein) is a 26KD periplasmic immunogenic protein which was simultaneously identified by three nonrelated research groups as an immunodominant antigen in infected cattle, sheep, goats, and humans [17], [19], [27], [35]. Use of an indirect ELISA to detect antibodies in brucellosis patients (n = 20) and uninfected controls (n = 35) yielded a sensitivity of 0.9 and specificity of 0.91 (not shown). Another serodiagnostic protein for both humans and goats was Protease DO, also designated as HtrA [31]. Use of an indirect ELISA to BMEI1330 yielded a sensitivity of 0.84 and a specificity of 0.99. Thus, the ELISA data were consistent with values determined using immunostrips and with the proteome array data. There are 11 antigens exclusively useful for human brucellosis diagnosis and 16 antigens exclusively for goats. Most of these are membrane proteins, lipoproteins, transporter proteins, proteins with signal peptide and proteins related to pathogenicity. We also identified 8 common cross-reactive antigens for both humans and goats, and 12 exclusively for humans and 23 for goats (**Fig. 6**, **Table 2**).

**Table 2:** Cross-reactive *Brucella melitensis* antigens for humans and goats.

	*B. melitensis*	Product Name
**Cross- reactive for both Humans and Goats**	BMEI1079	Lipoprotein NlpD
	BMEII0154	Flagellar motor protein
	BMEI0613	Protease Do
	BMEI1073	Glucose-inhibited division protein A
	BMEII0497	Enoyl-CoA hydratase/3-hydroxyacyl-CoA dehydrogenase
	BMEI0063	Hypothetical membrane spanning protein
	BMEII0010	Hypothetical membrane associated protein
	BMEI0847	Protein-export membrane protein
**Cross- reactive for Humans only**	BMEI0179	Hypothetical protein transporter
	BMEI2053	
	BMEI1646	Acriflavin resistance protein E
	BMEI1471	4-Amino-4-deoxychorismate lyase
	BMEI1236	Hypothetical exported proline-rich protein
	BMEI1692	Flagellar protein FlgJ
	BMEII0571	Acetolactate synthase IolD
	BMEII0793	Multidrug resistance efflux pump
	BMEI0123	Peptidyl-prolyl cis-trans isomerase
	BMEII0019	Stomatin like protein
	BMEII1118	Multidrug resistance protein A
	BMEI0848	Probable carnitine operon oxidoreductase CaiA
**Cross-reactive for Goats only**	BMEI1989	Phosphate-binding periplasmic protein periplasmic oligopeptide-binding protein precursor
	BMEII0735	
	BMEI0263	Leu-, Ile-, Val-, Thr-, and Ala-binding protein precursor
	BMEI1792	Hypothetical protein
	BMEII0381	Acriflavin resistance protein E
	BMEI0475	Cytochrome C1
	BMEI0053	Cation-transporting ATPase PacS
	BMEII1111	Hypothetical protein
	BMEI0443	Hypothetical protein
	BMEI1439	Chromosome segregation protein SMC2
	BMEI0340	Peptidoglycan-associated lipoprotein Omp16
	BMEI1954	ABC transporter substrate binding protein
	BMEII0678	Lipoyltransferase
	BMEI1334	Cytochrome C-type biogenesis protein CycH
	BMEI1280	Hypothetical cytosolic protein
	BMEI0749	DNA-directed RNA polymerase beta subunit
	BMEII0029	Type IV secretion apparatus protein VirB5
	BMEI1000	Hypothetical protein
	BMEI0821	Hypothetical protein
	BMEII0852	Succinoglycan biosynthesis transport protein
	BMEI1060	DsbA Protein-disulfide isomerase
	BMEI1428	Ribonuclease III
	BMEII0151	Flagellar Mring protein FliF

## Discussion

Here we report a large scale analysis showing that the humoral immune responses against *B. melitensis* protein antigens differ between humans naturally infected by consuming *Brucella melitensis*-contaminated, unpasteurized goat's milk products, and goats experimentally infected with *B. melitensis* by conjunctival instillation. These observations show that a natural reservoir host and the accidental human host have fundamentally different immune responses against this zoonotic pathogen. These data have implications for the practical development of diagnostics and reflect basic differences in host pathogen interactions and disease pathogenesis.

In addition, we demonstrate that a systematic, genome-wide analysis proved to identify protein antigens recognized by humans and animals not previously identified using Western blot or genomic library immunoscreening. Further, by virtue of being found to react with antibodies, the protein array technology is able to provide strong evidence of the comprehensive set of proteins expressed *in vivo* within a mammalian host by *B. melitensis*. As with our published experience with viral, bacterial and protozoal genomes expressed using protein microarray technology [44], [49], [50], [58], [64]–[66], conformation-dependent epitopes seem not to present problems with data interpretation or comprehensiveness of antigen discovery. This is likely because the polyclonal antibody response to protein antigens after infection detects both linear and 3-dimensional epitopes. The *B. melitensis* proteins placed onto the array, while expressed heterologously in a bacterial system, likely reflect a mix of conformationally correct as well as misfolded epitopes both of which are capable of binding specific antibodies.

Serological diagnosis of both human and animal brucellosis can suffer from the inability to distinguish new from previous infection (in the case of humans [1]) and differentiation of vaccination from new infection (in the case of animals [8]). In the absence of known exposure history in endemic regions, there is the possibility of mistaken diagnosis and overtreatment [10]. Current assays are based primarily on identification of antibodies to LPS in patient serum. Since *Brucella* LPS is cross-reactive with several other species, including *E. coli* O157:H7, *Yersinia enterocolitica* O9, and *Francisella tularensis* (although the clinical presentations of infectious caused by these agents are quite different), identification of diagnostic protein antigens may facilitate the development of more specific serodiagnostic assays [21], [67], [68]. The top 5 serodiagnostic antigens discovered using the protein microarrays had sensitivity and specificity of 95% and 96%, better than that of the 13 antigens on the immunostrips (94% and 89%), which in turn was roughly comparable to that of smooth LPS-based tests used in the Rose Bengal, lateral flow, and ELISA formats. In the present study however, the sensitivity of the serodiagnostic protein antigens could not be compared to that of the Rose Bengal test because we did not confirm any brucellosis cases among Rose Bengal negative patients by culture.

One interesting finding of this study was the difference in background reactivity to *B. melitensis* proteins in uninfected individuals from endemic vs. non-endemic areas. In Peru, control subjects tended to have higher background reactivity to *Brucella* antigens, compared to US control subjects (Fig. 3a). Consideration of these differences would be important for the development of diagnostic assays intended for use in both endemic and non-endemic regions of the world. The degree of variability between subjects differs depending on the infection and the results for *Brucella* reported here are similar to those that we obtained from patients with melioidosis [64], [65] and Lyme disease.

Our results with the *B. melitensis* proteome array represent the first large-scale analysis of *B. melitensis* proteins that are immunogenic in the context of naturally acquired human infections. In the case of the present study, the identified risk factor for human infection was ingestion of *B. melitensis*-contaminated, unpasteurized goat's milk products. In other epidemiological contexts, *B. melitensis* can be contracted by direct exposure to infected animals, not only goats, but also sheep and cattle [1], [3]. Further, we compared the set of proteins identified using human patient sera with the set that was immunogenic in the animal reservoir for zoonotic disease, the goat. Two proteins, BMEI0536 (Bp26) and BMEI1330 (HtrA/DegP), were immunogenic in the context of both infections. These results are in good agreement with previous reports on these antigens from other *Brucella* spp. Roop et al. [31] showed that HtrA was recognized by serum from goats, cattle and mice experimentally infected with *B. abortus* and by serum of dogs infected with *B. canis*. HtrA/DegP is a periplasmic serine protease that contributes to survival following stresses including oxidative damage. Bp26 has been proposed as a diagnostic antigen for detection of *B. melitensis* infection in sheep and *B. abortus* in cattle [17], [19]. The Omp16 lipoprotein (BMEI0340), originally identified as an immunogenic protein of *B. abortus* [32], was recognized by patient sera, but was found to be reactive in both infected and uninfected goats. Our results differ from those of Letesson et al., who reported no reactivity of uninfected goats to Omp16 [25]. This difference may reflect exposure of goats used in our study to other pathogens or to environmental bacteria expressing a cross-reactive antigen. The identification of known immunogenic *Brucella* proteins on the proteome array provided confirmation that our approach could identify both known and novel immunogenic proteins.

In addition to these well-characterized antigens, our study identified several novel serodiagnostic antigens specific for human *B. melitensis* patients (Table 1). These included BMEI1439 (SMC), an ATPase shown in other bacterial species to be involved in condensation and segregation of replicating chromosomes [29]. Further, the bacterial cell envelope proteins VirB8 (BMEII0032), DsbA (BMEI1060), and an uncharacterized transporter (BMEI1890), were immunogenic in patients. Three metabolic enzymes, acetyl coA carboxylase, (BMEI0503), Acetyl CoA carboxylase (BMEI0039 and 2-oxoglutarate dehydrogenase (BMEI0141) also represent novel serodiagnostic antigens for human brucellosis. The finding that these proteins are immunogenic suggests that they are expressed during *B. melitensis* infection of humans.

A group of 16 antigens was found to be serodiagnostic for goats, but not humans (Table 1). These included 7 predicted transporters, as well as a zinc-binding protein and two binding proteins for calcium: Asp24 (BMEI0668) [26], and a second, uncharacterized calcium-binding protein (BMEI2053). The chaperonin GroES, shown to be seroreactive in a patient suffering from *B. suis* infection [20], was serodiagnostic in goats, as well as Bscp31 (BMEI0796), a well-known seroreactive protein [28].

The differences in the patterns of goat and human seroreactivity to *B. melitensis* proteins could be explained in several different ways. First, the group of seroreactive proteins in goats and humans gives some insight into the metabolic pathways expressed during infection in these hosts. The large number of immunogenic proteins with predicted function in nutrient uptake suggests that *B. melitensis* utilizes peptides and amino acids for growth during infection. Three serodiagnostic *B. melitensis* proteins (BMEI0340, BMEI0141 and BMEI0178), have been found to be upregulated during intracellular infection by *B. suis* or *B. abortus* [18], [24], suggesting that they may play a role in adaptation to intracellular life in the host. Second, the immunogenetics of immune responses to *B. melitensis* infection may be related to species differences between humans and goats but further comment on this possibility is limited by the lack of available data. Third, while humans in this study were thought to have been infected by ingestion of infected goat's milk products containing a small inoculum, the goats were infected with a high dose (10^7^ CFU) of *B. melitensis* strain 16M via the conjunctiva. Both dose and route of inoculum may have contributed to the differential antigen recognition between goats and humans, and, in fact, among humans as well. Finally, while it seems unlikely that the different patterns of immune response variation between goats and humans were due to protein expression differences by different strains of *B. melitensis*, this possibility must be considered as well.

The results presented here represent an analysis of 1406 proteins of 3198 predicted proteins in the *B. melitensis* 16M genome. Of these 1406 proteins, we only observed less than two fold enrichment of serodiagnostic antigens in the 1009 selected versus randomly selected antigens (not shown). Completion of the proteome array is currently underway, which will allow a more complete genome-level analysis of all immunogenic *B. melitensis* proteins. The subset of diagnostic antigens identified here provided an initial estimated accuracy rate of 95% for diagnosis of human cases and it is likely that this set of antigens will form the basis of a new and accurate serodiagnostic assay for human brucellosis. The clinical and veterinary utility of the protein antigens discovered in this study for diagnosis of acute and chronic brucellosis awaits validation in prospective studies in endemic regions.
